# Modulation of Human Adipose Stem Cells’ Neurotrophic Capacity Using a Variety of Growth Factors for Neural Tissue Engineering Applications: Axonal Growth, Transcriptional, and Phosphoproteomic Analyses In Vitro

**DOI:** 10.3390/cells9091939

**Published:** 2020-08-21

**Authors:** Katharina M. Prautsch, Alexander Schmidt, Viola Paradiso, Dirk J. Schaefer, Raphael Guzman, Daniel F. Kalbermatten, Srinivas Madduri

**Affiliations:** 1 Department of Plastic, Reconstructive, Aesthetic and Hand Surgery, University Hospital Basel, Spitalstrasse 21, 4021 Basel, Switzerland; katharina.prautsch@unibas.ch (K.M.P.); Dirk.Schaefer@usb.ch (D.J.S.); Daniel.Kalbermatten@usb.ch (D.F.K.); 2 Department of Pathology, University Hospital Basel, Hebelstrasse 20, 4021 Basel, Switzerland; viola.paradiso@usb.ch; 3 Department of Biomedical Engineering, University of Basel, Gewerbestrasse 14, 4123 Allschwil, Switzerland; 4 Biozentrum, University of Basel, Klingelbergstrasse 50/70, 4056 Basel, Switzerland; alex.schmidt@unibas.ch; 5 Department of Biomedicine, University Hospital Basel, Hebelstrasse 20, 4021 Basel, Switzerland; Raphael.Guzman@usb.ch; 6 Department of Neurosurgery, University Hospital Basel, Spitalstrasse 21, 4021 Basel, Switzerland

**Keywords:** human adipose stem cells, neurotrophic factors, peripheral nerve injuries, axonal regeneration, Schwann cells, ex vivo stimulation, dorsal root ganglion

## Abstract

We report on a potential strategy involving the exogenous neurotrophic factors (NTF) for enhancing the neurotrophic capacity of human adipose stem cells (ASC) in vitro. For this, ASC were stimulated for three days using NTF, i.e., nerve growth factor (NGF), brain-derived neurotrophic factor (BDNF), neurotrophin 3 (NT3), NT4, glial cell-derived neurotrophic factor (GDNF), and ciliary neurotrophic factor (CNTF). The resulting conditioned medium (CM) as well as individual NTF exhibited distinct effects on axonal outgrowth from dorsal root ganglion (DRG) explants. In particular, CM derived from NT3-stimulated ASC (CM-NT3-ASC) promoted robust axonal outgrowth. Subsequent transcriptional analysis of DRG cultures in response to CM-NT3-ASC displayed significant upregulation of STAT-3 and GAP-43. In addition, phosphoproteomic analysis of NT3-stimulated ASC revealed significant changes in the phosphorylation state of different proteins that are involved in cytokine release, growth factors signaling, stem cell maintenance, and differentiation. Furthermore, DRG cultures treated with CM-NT3-ASC exhibited significant changes in the phosphorylation levels of proteins involved in tubulin and actin cytoskeletal pathways, which are crucial for axonal growth and elongation. Thus, the results obtained at the transcriptional, proteomic, and cellular level reveal significant changes in the neurotrophic capacity of ASC following NT3 stimulation and provide new options for improving the axonal growth-promoting potential of ASC in vitro.

## 1. Introduction

Peripheral nerve injuries with loss of functional tissue are a common clinical problem whereby patients suffer lifelong sensory-motor deficits in spite of advanced microsurgical procedures and functional follow-ups [1,2,3,4]. However, nerve injuries have no viable alternative to match the regenerative level of the gold standard (autologous nerve grafts) [3,4,5]. Thus, the need for nerve tissue engineering approaches emerged.

The process of timely and effective nerve regeneration is primarily influenced by the regenerative microenvironment, which comprises sustained growth factor release, permissive extracellular matrix scaffolds, and Schwann cells facilitating the axonal guidance [6,7]. Therefore, the unsatisfactory clinical outcomes obtained so far can be attributed to the failed maintenance of a permissive microenvironment within the niche of the regenerating nerve tissue. Nerve tissue engineering strategies aim at sustaining an effective microenvironment for functional nerve regeneration [3,4,8]. Within this context, Schwann cells (SC) play a key role [9].

Following injury, SC initiate Wallerian degeneration, express a wide range of cytokines and neurotrophic factors (NTF), and support axonal path finding [10,11,12,13]. SC express p75, TrkA, TrkB, TrkC, and GFRα receptors [14,15,16,17,18,19,20,21], which are responsive for various growth factors and rapidly establish paracrine signaling in response to endogenous growth factors [8,10,12,13]. Several studies proved the beneficial effects of transplanted SC for nerve repair and axonal regeneration [22,23,24,25]. However, the harvest of autologous SC requires a painful nerve biopsy for the patient, followed by rather challenging culture conditions, which result in significant treatment delay [26,27]. Therefore, stem cells gained a growing interest as a viable alternative [28,29].

Adipose-derived stem cells (ASC) can be harvested in abundance from adipose tissue [30,31,32], grow fast, and have a low immunogenic profile [33,34]. They secrete an array of neurotrophic factors, chemokines, and miRNA [35,36,37,38] and can be differentiated into SC-like cells (SCLC) in order to improve their neurotrophic properties [35,36,38,39]. ASC differentiation into SCLC requires extended stimulation involving a complex array of exogenous factors over up to three weeks, indicating a major hurdle to their clinical use. Moreover, it was demonstrated that SCLC rapidly revert to fibroblast cell-like characteristics after the withdrawal of the differentiation medium. Therefore, their beneficial effects remain questionable [40,41,42,43], and there is a clear need for developing simple and effective strategies for enriching the neurotrophic properties of ASC [44].

Following injury, a wide range of NTF, which can be attributed to neuronal and non-neuronal sources [45], are rapidly overexpressed [7,46,47]. Thus, the therapeutic targets of severed nerves, i.e., neurons and glial cells, require neurotrophic support and structural guidance for repair and regeneration [48]. Although peripheral nervous system is capable of spontaneous regeneration due to the intrinsic regenerative capacity, long-gap and chronic nerve injuries, in particular, require exogenous trophic and topographical support for effective regeneration [49]. However, recent studies showed the deleterious effects of using large number of biological factors for promoting axonal regrowth while the therapeutic benefits are being ineffective [50]. Thus, the need for effective and viable alternatives such as cell therapies emerged [28]. Cell with improved trophic capacity for addressing the complex needs of severed nerves would circumvent the side effects associated with application of large number of exogenous factors [29]. ASC and SCLC were shown to support nerve regeneration by releasing a variety of biological factors with mitotic and neuritogenic activity, although neurotrophic capacity of ASC is still inferior to SC [51]. Growth factors and related receptors play key role in the SC lineage and their autocrine survival loop [52]. Interestingly, ASC were found to express most of the receptors for NTF [35,37,53,54,55,56] suggesting that ASC may operate like SC in response to NTF stimulation. Within this context, we recently demonstrated enhanced neurotrophic capacity of ASC in response to nerve growth factor (NGF) stimulation in vitro and in vivo [51].

Given these facts, we hypothesized that NTF may have a fundamental role in the neurotrophic capacity of the stem cells, which is related to SC lineage, which in turn is an important topic to basic neurobiology (SC lineage) and nerve tissue engineering. Therefore, it is fundamental to understand the role of variety of NTF individually on the fate of ASC at cellular and molecular levels. However, studies elucidating the influence of NTF on ASC biology (neurotrophic capacity) are largely missing, thus there is a great need for shedding light on this key topic [51].

Therefore, in the present study, we have evaluated different NTF representing neurotrophins, i.e., NGF, brain-derived neurotrophic factor (BDNF), neurotrophin 3 (NT3), and NT4; the glial cell-derived neurotrophic factor (GDNF) family of ligands, i.e., GDNF, and neurotrophic cytokines, i.e., CNTF [7,47,57] for ex vivo stimulation of ASC. Subsequently, we assessed the axonal growth-promoting capacity of a conditioned medium (CM) derived from NTF-stimulated ASC as well as individual NTF and found significant effects on axonal outgrowth. Furthermore, molecular changes taking place in the stimulated ASC as well as in the treated DRG explant cultures were studied by transcriptional and phosphoproteomic analyses.

## 2. Materials and Methods

### 2.1. Isolation and Culture of Human Adipose Stem Cells (ASC)

Adipose tissue was obtained from healthy patients undergoing elective liposuction or abdominoplasty. All patients had signed an informed consent form prior to the surgery. The isolation of ASC was performed under sterile conditions according to a well-established protocol [39]. The fat tissue was cleaned from erythrocytes by rinsing with 0.01M phosphate-buffered solution (PBS) and centrifugation, then minced and digested with 0.1% (*w*/*v*) Type I Collagenase (Gibco Life Technologies, Basel, Switzerland, Cat. No. 17100017) for 3 h at 37 °C and centrifuged for 5 min at 1500 rpm and 4 °C. The resulting pellet was resuspended in growth medium (GM), i.e., Dulbecco’s Modified Eagle’s Medium (DMEM, Gibco, Cat. No. 41965039) supplemented with 10% fetal bovine serum (FBS) (PAN-Biotech, Aidenbach, Germany, EU-approved, Cat. No. P40-47500) and 1% penicillin/streptomycin (BioConcept, Allschwil, Switzerland, Cat. No. 4-01F00-H). Extracted ASC were seeded at a density of 3000 cells/cm2 and cultured at 37 °C with 5% CO_2_ in a humid atmosphere, with GM being changed every 72 h. Cell passage was performed at 90% confluence by using 0.25% trypsin—EDTA (BioConcept, Cat. No. 5-51F00-H) and resulting cells at passage 2 (P2) or 3 (P3) were used for the experiments.

### 2.2. Human ASC Characterization

P2 human ASC were seeded on 24-well plates for characterization. Cells were fixed in 4% paraformaldehyde (PFA) at room temperature (RT) for 10 min and permeabilized and blocked in PBS containing 0.1% Triton X-100 and 1% normal goat serum (NGS) (i.e., dilution buffer) for 20 min at RT. ASCs were incubated overnight at 4 °C with the human mesenchymal stromal cell markers monoclonal mouse anti-CD44 (1:1000), monoclonal mouse anti-CD90 (1:200), monoclonal mouse CD105 (1:200), monoclonal rabbit anti-CD29 (1:100), and polyclonal rabbit anti-CD45 (1:500) (Abcam, Cambridge, UK, Cat. No. ab93758). Cells were then washed in PBS and incubated for 1 h at RT with the secondary antibody goat anti-mouse Alexa Fluor 488 (1:500, Abcam, Cat. No. ab150109) and goat anti-rabbit Alexa Fluor 488 (1:500, Abcam, Cat. No. ab150061), and Hoechst 33258 nuclear staining (1:1000, Sigma-Aldrich, Buchs, Switzerland, Cat. No. 94403). Subsequently, digital images were acquired at 20× magnification (numerical aperture 0.45) using a Nikon Eclipse Ti2 inverted fluorescent microscope (Nikon Eclipse Ti_2_-E, -E/B, Nikon Corporation, Tokyo, Japan) and a Nikon DS-Qi2 camera (Nikon Corporation, Japan). The images were automatically stitched by the Nikon NIS-Elements AR image analysis software (NIS-Elements AR Analysis 5.11.00 64-bit, Laboratory Imaging, spol. s.r.o., Praha, Czech Republic). The results are shown in Figure S1.

### 2.3. Isolation of Chicken Embryonic Dorsal Root Ganglia (DRG)

Fertilized chicken eggs were ordered from Gepro Geflügelzucht AG (Flawil, Switzerland). The eggs were shipped at ambient temperature and incubated at 37.8 ± 0.2 °C under 100% relative humidity for 10 days (E10). After incubation, the eggs were wiped with 70% ethanol and embryos were collected under a laminar airflow cabinet. Dissection of the E10 embryos was performed with the help of a stereomicroscope following a standard dissection protocol [29,58,59]. DRG were explanted from the lumbar part of the spine and stored in GM on ice until cell culture.

### 2.4. Quantitative Measurement of NT3 by ELISA

CM medium from the NT-3 stimulated ASC and unstimulated ASC was collected. ELISA kit was obtained from R&D systems (Cat. No. dy267 and DY008) and analysis was run as per the protocol provided by manufacturer. Briefly, 96-well plates were incubated with 100 μL of the respective capture antibody overnight at 4 °C. Plates were then blocked for a minimum of 1 h at RT by adding 300 μL of reagent diluent to each well. Subsequently, 100 μL of sample or standards were added per well and incubated 2 h at RT. Standards were prepared using serial dilutions. Plates then underwent consecutive incubations at RT for each 20 min, with 100 μL of Streptavidin-HRP A and with 100 μL of Substrate Solution. Finally, 50 μL of Stop Solution was added to each well. Optical density of each well was determined immediately, using the Synergy H1 micro-plate reader (BioTek Instruments, Winooski, VT, USA) set to 450 nm. For wavelength correction, readings at 540 nm were subtracted from the readings at 450 nm. The program used was Gen5 2.05 (BioTek Instruments, Winooski, VT, USA).

### 2.5. Experimental Design for the Axonal Outgrowth Assay

**Figure 1:**
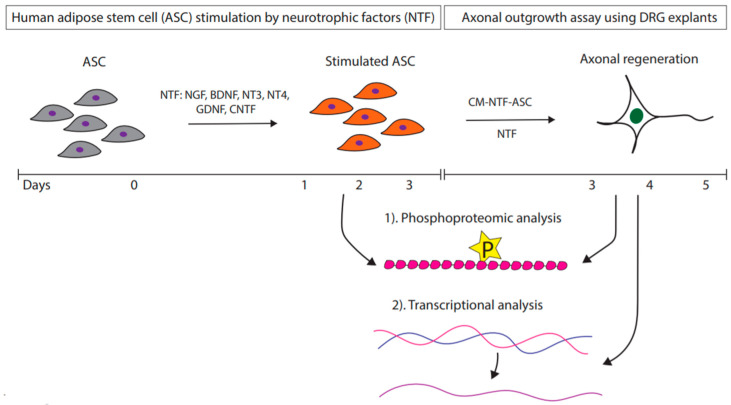
Ex-vivo stimulation of adipose stem cells using different neurotrophic factors. Key. NTF: neurotrophic factors; NGF: nerve growth factor; BDNF: brain-derived neurotrophic factor; NT3: neurotrophin 3; NT4: neurotrophin 4; GDNF: glial cell-derived neurotrophic factor; CM-NT3-ASC: conditioned medium derived from adipose stem cells; DRG: dorsal root ganglion.

As illustrated in Figure 1, freshly split ASC seeded at 50–60% confluency in 24-well plates with 500 μL of volume were stimulated by rhNGF (NGF-ASC), rhGDNF (GDNF-ASC), rhBDNF (BDNF-ASC), rhCNTF (CNTF-ASC), rhNT3 (NT3-ASC), or rhNT4 (NT4-ASC) or with no neurotrophic factor (ASC) for 72 h. The resulting conditioned medium (CM), i.e., CM-NGF-ASC, CM-GDNF-ASC, CM-BDNF-ASC, CM-CNTF-ASC, CM-NT3-ASC, CM-NT4-ASC, and CM-ASC, were subsequently used in the DRG assays. As controls, culture conditions with growth media supplemented with the respective NTF, i.e., NGF alone (NGF), GDNF alone (GDNF), BDNF alone, (BDNF), CNTF alone (CNTF), NT3 alone (NT3), NT4 alone (NT4), and the growth medium without NTF-supplementation (GM), were used. For all conditions, neurotrophic factors were applied at 10 ng/mL, based on preliminary experiments [60]. Neurotrophic factors were purchased from R&D Systems Inc. (Minneapolis, MN, USA; Art. No. 256-GF, 212-GD, 248-BDB, 257-NT, 267-N3, 268-N4). For the axonal outgrowth assay, DRG explants were cultured at a density of one per well onto 24-well plates in 500 μL of the respective CM. Cultures were maintained in a humid atmosphere at 37 °C and 5% CO_2_ for 48 h and images were captured at 5× and 10× magnification. In total, there were three independent experiments were performed, resulting in a total of 12 DRG explant cultures for each experimental condition.

### 2.6. Immunocytochemistry of DRG Cultures

After 48 h, brightfield images with a phase contrast of DRG-explants were taken at 5× magnification using a Zeiss Axio Vert.5A1 inverted fluorescent microscope (Carl Zeiss AG, Oberkochen, Germany) and a Zeiss AxioCam MRc camera. DRG explants were then fixed in 4% PFA at RT for 10 min and permeabilized and blocked in PBS containing 0.1% Triton X-100 and 1% bovine serum albumin (BSA) (i.e., the dilution buffer) for 20 min at RT. For immunocytochemistry, the cultures were incubated overnight at 4 °C with the primary antibody monoclonal mouse anti-β-Tubulin III (1:1000, Sigma-Aldrich, Buchs, Switzerland, Cat. No. T8578) for regenerating axons. The cultures were then washed in PBS and incubated for 1 h at RT with the secondary antibody sheep anti-mouse Cy3 (1:500, Sigma Aldrich, Cat. No. C2181) and Hoechst 33258 nuclear staining (1:1000, Sigma Aldrich, Cat. No. 94403). Digital images of the stained specimens were acquired at 15× magnification (numerical aperture 0.45) using a Nikon Eclipse Ti_2_ inverted fluorescent microscope and a Photometrics prime 95B 25 mm camera (Teledyne Photometrics, Tucson, AZ, USA). The images were automatically stitched by the Nikon NIS-Elements AR image analysis software.

### 2.7. Quantitative Analysis of Axonal Outgrowth

DRG explant cultures were measured for axonal length and axonal area with an automated and standardized analysis mask created by using the Nikon NIS-Elements AR image analysis software.

### 2.8. Quantitative RT-PCR for Regeneration-Associated Genes (RAG) in DRG

DRG explants grown for 48 h in an NT3-stimulated ASCs conditioned medium (CM-NT3-ASC), unstimulated ASCs conditioned medium (CM-ASC), NT3-supplemented GM (NT3), and GM only (GM) were evaluated for the upregulation of the regeneration-associated genes STAT-3 and GAP-43. β-Actin was used as the housekeeping gene. A total of 500 ng RNA per sample was converted into cDNA following the protocol for the SuperScript™ IV VILO™ Master Mix with ezDNase™ Enzyme (Invitrogen™, Cat. No. 11766050) and using the SimpliAmp™ Thermocycler (Applied Biosystems™, CA, USA, Cat. No. A24811 by Life Technologies, Thermo Fisher Scientific AG, Basel, Switzerland). Amplification was performed with the QuantStudio™ 3 Real-Time PCR Systems (Applied Biosystems, Thermo Fisher Scientific AG) using a Fast Start Universal SYBR Green Master Mix (Roche, Cat. No. 04 913 850 001). Twenty nanograms of cDNA were used per well. The primers used are listed in Figure 4B and were designed for the species Gallus gallus. All reactions were performed using the same conditions: 50 °C for 2 min, 95 °C for 10 min; 40 cycles at 95 °C for 15 s, 60 °C for 1 min. The experiment was repeated three times and all experimental samples were assayed in triplicate. A negative control (RNase-free water) was always included. The expression levels of STAT-3 and GAP-43 were determined in relation to the β-actin RNA levels.

### 2.9. Phosphoproteomic Analysis

Freshly split ASC seeded at 50–60% confluency in 75-cm^2^ culture flasks were either stimulated by NT3 (NT3-ASC) or had no growth factor (ASC) for 72 h. After incubation, cells were detached by scratching and subjected to two cycles of centrifugation for 5 min at 1500 rpm and 4 °C and PBS washing. After the last wash, the PBS was discarded and the cell pellet was immediately transferred to −80 °C for storage. For the DRG samples, the same experimental groups and culture conditions as for the qRT-PCR experiments were applied. After 48 h of incubation in the respective conditioned medium, the DRG explants were subjected to the same treatment as described above for ASC, except for centrifugation, which was performed at 900 rpm. A protein yield of at least 100 μg per experimental condition was required for the phosphoproteomic analysis. The experiments were repeated three times, resulting in three independent samples for ASC and DRG explants.

Cells were lysed in 8M Urea (Sigma-Aldrich, Buchs, Switzerland), 0.1M ammonium bicarbonate in the presence of phosphatase inhibitors (Sigma P5726&P0044) using strong ultrasonication, i.e., 10 cycles, 30 s on/off, (Bioruptor, Seraing, Belgium). The protein concentration was determined by a BCA assay (Thermo Fisher Scientific, Basel, Switzerland) using a small sample aliquot. Two hundred fifty micrograms of proteins were digested as previously described (PMID: 27345528), reduced with 5 mM TCEP for 60 min at 37 °C and alkylated with 10 mM chloroacetamide for 30 min at 37 °C. After diluting the samples with 100 mM ammonium bicarbonate buffer to a final urea concentration of 1.6 M, the proteins were digested by incubation with sequencing-grade modified trypsin (1/50, *w*/*w*; Promega, Madison, WI, USA) overnight at 37 °C. After acidification using 5% TFA, the peptides were desalted on C18 reversed-phase spin columns according to the manufacturer’s instructions (Macrospin, Harvard Apparatus, Holliston, MA, USA) and dried under a vacuum.

Peptide samples were enriched for phosphorylated peptides using Fe(III)-IMAC cartridges on an AssayMAP Bravo platform as recently described (PMID: 28107008). The setup of the μRPLC-MS system was as described previously (PMID:27345528). Chromatographic separation of peptides was carried out using an EASY nano-LC 1200 system (Thermo Fisher Scientific), equipped with a heated RP-HPLC column (75 μm × 37 cm) packed in house with 1.9 μm C18 resin (Reprosil-AQ Pur, Dr. Maisch). Aliquots of 1 μg total peptides were analyzed per LC-MS/MS run using a linear gradient ranging from 95% solvent A (0.1% formic acid) and 5% solvent B (80% acetonitrile, 19.9% water, 0.1% formic acid) to 30% solvent B over 90 min at a flow rate of 200 nL/min. Mass spectrometry analysis was performed on a Tribrid Orbitrap Lumos mass spectrometer equipped with a nanoelectrospray ion source (both Thermo Fisher Scientific). Each MS1 scan was followed by high-collision dissociation (HCD) of the 10 most abundant precursor ions with dynamic exclusion for 30 s. The total cycle time was approximately 1 s. For MS1, 1e6 ions were accumulated in the Orbitrap cell over a maximum time of 100 ms and scanned at a resolution of 120,000 FWHM (at 200 *m*/*z*). MS2 scans were acquired at a target setting of 100%, accumulation time of 54 ms, and a resolution of 30,000 FWHM (at 200 *m*/*z*). Singly charged ions and ions with unassigned charge state were excluded from triggering MS2 events. The normalized collision energy was set to 30%, the mass isolation window was set to 1.4 *m*/*z,* and one microscan was acquired for each spectrum.

The acquired raw files were imported into the Progenesis QI software (v2.0, Nonlinear Dynamics Limited), which was used to extract peptide precursor ion intensities across all samples by applying the default parameters. The MGF files generated were searched against a human/chicken database containing the usually observed contaminants and a total of 41,592 human protein sequences [61]/55,856 chicken protein sequences [62] using MASCOT and the following search criteria: full tryptic specificity was required (cleavage after lysine or arginine residues, unless followed by proline); three missed cleavages were allowed; carbamidomethylation (C) was set as the fixed modification; oxidation (M) and phosphorylation (STY) were applied as variable modifications; mass tolerance of 10 ppm (precursor) and 0.02 Da (fragments). The database search results were filtered using the ion score to set the false discovery rate (FDR) to 1% on the peptide and protein level, respectively, based on the number of reverse protein sequence hits in the datasets. The relative quantitative data obtained were normalized and statistically analyzed using our in-house script as above (PMID:27345528). The complete list of quantified phosphorylation sites is provided as supplemental data (Tables S1 and S2). All raw data associated with this manuscript are publicly available.

### 2.10. Data Availability

The mass spectrometry proteomics data have been deposited to the ProteomeXchange Consortium via the PRIDE [PubMed ID: 30395289] partner repository with the dataset identifier PXD019015 and 10.6019/PXD019015. (Reviewer account details: Username: reviewer59495@ebi.ac.uk, Password: rUv034jQ.)

### 2.11. Statistical Analysis

Data were analyzed by one-way analysis of variance (ANOVA) following the Bonferroni procedure with post hoc multiple comparisons using SPSS (version 15.0; SPSS, Chicago, IL, USA). Values of *p* < 0.05 were considered significant.

## 3. Results

### 3.1. Human ASC Characterization

The phenotype of ASC was quantitatively characterized using representative images and ImageJ for analysis. The cells were found to be positive for mesenchymal marker CD29 at 87%, CD44 at 88%, CD90 at 92%, and CD105 at 93%, and negative for hematopoietic marker CD45 (Figure S1). Further, NT3-stimulated ASC also displayed similar expression pattern of mesenchymal stem cell (MSC) markers including S100 of SC at 96%, but no expression was observed for other specific markers, i.e., GFAP and p75 (Figure S2).

### 3.2. Distinct Effects of NTF on Axonal Outgrowth

**Figure 2:**
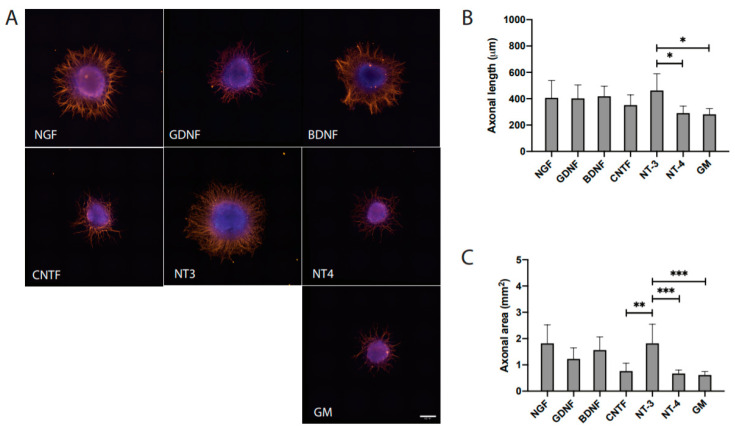
Distinct effects of individual NTF on axonal outgrowth. (**A**) Microphotographs of dorsal root ganglion (DRG) explants treated with NTF or growth medium alone (GM), (**B**) Quantitative measurements of axonal length (μm), and (**C**) Measurements of axonal area (μm^2^). The scale bar represents 500 μm. The bars represent mean ± SD of *n* = 12. Significant differences are indicated as * *p* < 0.05, ** *p* < 0.01 and *** *p* < 0.001.

As illustrated in Figure 1, various NTF were used for the stimulation of axonal growth. Interestingly, all the growth factors promoted considerable axonal outgrowth in comparison to GM. Notably, NT3 promoted significant axonal outgrowth (Figure 2A–C). Quantitative measurements of axonal length (in μm) from DRG explants treated with growth factors resulted in 413 ± 182 for NGF, 405 ± 116 for GDNF, 419 ± 73 for BDNF, 352 ± 74 for CNTF, 463 ± 121 for NT3, 291 ± 51 for NT4, and 282 ± 41 for GM (Table 1). Interestingly, the axonal growth pattern in response to various NTF treatments appeared to be distinctive. NGF promoted dense axonal growth without longer projections in contrast to GDNF, which resulted in relatively longer axonal projections without a branching effect. BDNF influenced axonal elongation as well as branching. Notably, NT3 promoted radially aligned axonal growth with relatively longer axonal projections. NT4 as well as CNTF resulted in only minimal axonal outgrowth (Figure 2). Quantitative measurements of the axonal area (in μm^2^) in DRG cultures amounted to 1.903 ± 1.239 for NGF, 1.252 ± 0.486 for GDNF, 1.566 ± 0.479 for BDNF, 0.766 ± 0.286 for CNTF, 1.819 ± 0.700 for NT3, 0.674 ± 0.123 for NT4, and 0.616 ± 0.125 for GM (Table 1).

### 3.3. Conditioned Medium Derived from Stimulated Stem Cells

**Figure 3:**
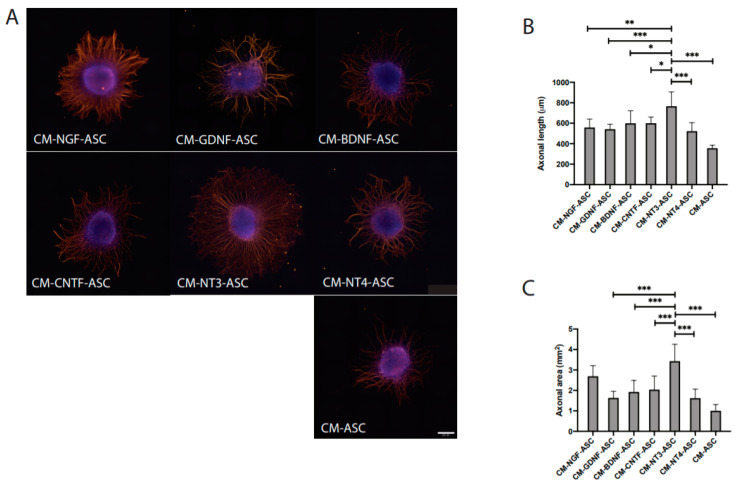
Neurotrophic potency of conditioned medium (CM) derived from NTF-stimulated ASC for supporting axonal regeneration. (**A**) Microphotographs of DRG explants treated with CM derived from NTF-stimulated ASC, (**B**) Quantitative measurements of axonal length (μm), and (**C**) Measurements of axonal area (μm^2^). The scale bar represents 500 μm. The bars represent mean ± SD of *n* = 12. Significant differences are indicated as * *p* < 0.05, ** *p* < 0.01, and *** *p* < 0.001.

The NTF-stimulated ASCs conditioned medium (CM) resulted in an enhanced axonal outgrowth in comparison to unstimulated ASC and NTF treatment alone (Figure 3A–C). Quantitative measurements of axonal length (in μm) amounted to 526 ± 87 for CM-NGF-ASC, 505 ± 75 for CM-GDNF-ASC, 598 ± 118 for CM-BDNF-ASC, 599 ± 58 for CM-CNTF-ASC, 765 ± 134 for CM-NT3-ASC, 522 ± 80 for CM-NT4-ASC, and 469 ± 99 for CM-ASC (Table 1). Significant differences were observed for CM-BDNF-ASC (*p* < 0.05), CM-CNTF-ASC (*p* < 0.001), CM-NT3-ASC (*p* < 0.001), and CM-NT4-ASC (*p* < 0.001). In particular, CM-NT3-ASC triggered significantly robust axonal outgrowth in comparison to all other treatment conditions. Quantitative measurements of axonal area (in μm^2^) resulted in 2.460 ± 0.586 for CM-NGF-ASC, 1.512 ± 0.334 for CM-GDNF-ASC, 1.918 ± 0.547 for CM-BDNF-ASC, 2.032 ± 0.643 for CM-CNTF-ASC, 3.423 ± 0.798 for CM-NT3-ASC, 1.617 ± 0.427 for CM-NT4-ASC, and 1.166 ± 0.456 for CM-ASC (Table 1). In agreement with the axonal length measurements, CM-NT3-ASC exhibited significantly higher axonal outgrowth area (Figure 3C). Notably, CM-NT3-ASC promoted numerous radially aligned axons, displaying longer projections than all other conditions (Figure 3A).

### 3.4. NT3 Content

Quantitative analysis of the CM resulting from NT3-stimulated ASC amounted to 1.1 ± 0.32 ng/mL, which is significantly higher than physiological concentration 25–40 pg/mL [63,64,65] and lower than the amount 10 ng/mL used for the experiment. In contrast, unstimulated ASC showed no detectable NT3.

### 3.5. Transcriptional Analysis of RAG in Treated DRG Explants

**Figure 4:**
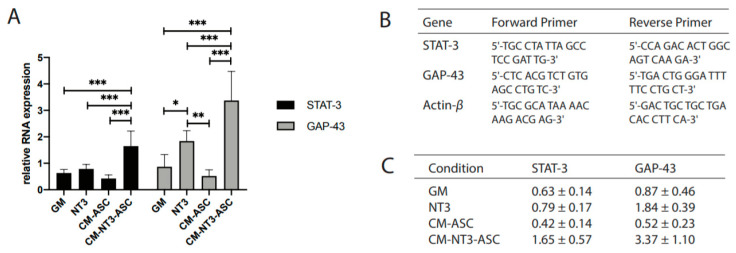
Transcriptional analysis of DRG explants treated with differential stimuli. **A**) Analysis of STAT-3 and GAP-43 mRNA expression by qRT-PCR, **B**) Primer sequences used for reverse transcription with polymerase chain reaction, and **C**) Quantitative measurements of mRNA expression. Expression values are relative to β-actin expression and are therefore in arbitrary units, shown as the mean ± SD of *n* = 9. Significant differences are indicated as * *p* < 0.05, ** *p* < 0.01, and *** *p* < 0.001.

Analysis by qRT-PCR for relative STAT-3 RNA levels resulted in Ct values of 1.65 ± 0.57 for CM-NT3-ASC, 0.42 ± 0.14 for CM-ASC, 0.79 ± 0.17 for NT3, and 0.63 ± 0.14 for GM (Figure 4C). Relative GAP-43 Ct RNA values showed 3.37 ± 1.10 for CM-NT3-ASC, 0.52 ± 0.23 for CM-ASC, 1.84 ± 0.39 for NT3, and 0.87 ± 0.46 for GM. Significant upregulation of the regeneration-associated genes STAT-3 and GAP-43 was observed for DRG explants treated with CM-NT3-ASC in comparison to all other conditions (Figure 4A–C).

### 3.6. Quantitative Phosphoproteomic Analysis

**Figure 5:**
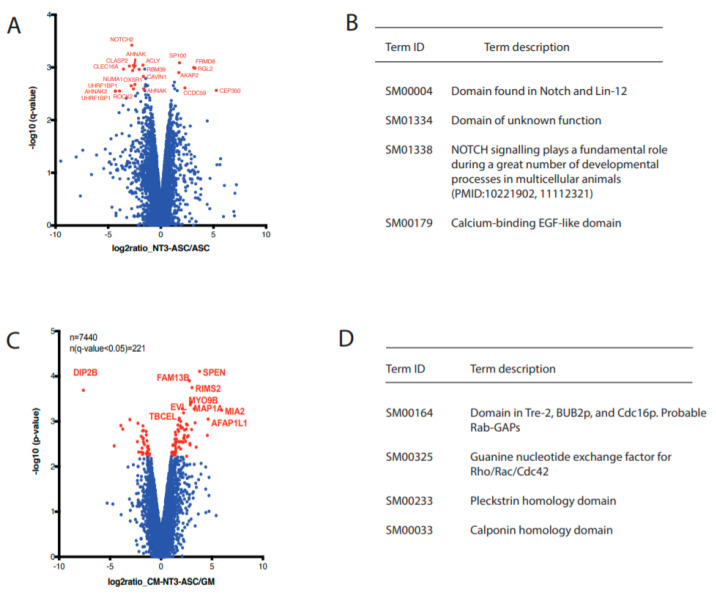
Quantitative phosphoproteomics analysis. (**A**) Volcano plots of phosphorylation site ratios and significance (*p*-value) obtained for NT3-stimulated ASC and unstimulated ASC comparisons. Significant hits with a *q*-value of less than 0.05 as computed by SafeQuant using paired t-test and Benjamini-Hochberg correction are indicated in red. The proteins associated with the 21 most significantly changing phosphorylation sites are also shown, (**B**) Significantly enriched terms (*p* < 0.05) found for the most significant hits using STRING, (**C**) Volcano plots of phosphorylation site ratios and significance (*p*-value) obtained for CM-NT3-ASC and GM comparisons. Significant hits with a *q*-value of less than 0.05 as computed by SafeQuant using paired *t*-test and Benjamini-Hochberg correction are indicated in red. The proteins associated with the 10 most significantly changing phosphorylation sites are also shown and (**D**) Significantly enriched terms (*p* < 0.05) found for the most significant hits using STRING.

To obtain a global view of the signaling events taking place upon growth factor treatment, a mass spectrometry-based phosphoproteomic analysis was performed. Two different experiments were carried out comparing NT3-ASC vs. ASC as well as DRG explant cultures treated with various stimuli conditions, i.e., CM-NT3-ASC, CM-ASC, NT3, and GM. All quantitative analyses were performed in triplicate, analyzing phosphopeptide-enriched samples using a label-free quantification of the LC-MS approach (PMID: 30867597). In total, 10,352 and 7440 phosphorylation sites were quantified for ASC and DRG explants, respectively (Figure 5, Figures S3 and S4, and Tables S1 and S2). ASC, in response to NT3 stimulation, exhibited the 21 most significantly changing phosphorylation sites (Figure 5A). This includes FRMD8 and NOTCH2, which are involved in regulated growth factors release and neural progenitor cell (NPC) maintenance and differentiation. Enrichment analysis of the altered phosphorylation sites further confirmed that the genes are associated with the chemokine signaling pathway and are significantly affected by NT3 stimulation (Figure 5B, Figure S3, and Table S1). These results further support the significantly enhanced axonal outgrowth in response to CM-NT3-ASC (Figure 3).

Subsequent comparison of DRG explants treated with CM-NT3-ASC vs. GM displayed the 10 most significantly changing phosphorylation sites (Figure 5C,D, Figure S3, and Table S2). Proteins associated with these sites include EVL, SPEN, and MAP1A, which are linked to growth cone formation and the polymerization, reorganization, and stabilization of axonal cytoskeletal proteins (i.e., tubulin and actin). An enrichment analysis further confirmed that tubulin and actin cytoskeletal pathway was significantly altered between these two conditions (Figure 5D), with elevated activity found for CM-NT3-ASC. The control (GM) DRG cells were also compared to the other treatment conditions, i.e., CM-ASC and NT3 (Figure S4); we found no robust changes. Most changes in the phosphoproteome were observed upon CM-NT3-ASC treatment, whereas treatment with NT3 alone or CM-ASC did not have a significant impact on the *p*-value frequency distribution (Figure S2A–C and Figure S4), indicating that the secretome of NT3-stimulated ASC is the trigger for the phosphorylation-based signaling events identified. Interestingly, these findings are consistent with the axonal outgrowth studies (Figure 3).

## 4. Discussion

Functional nerve regeneration is clinically challenging [5]. After injury, affected neurons and glial cells require neurotrophic support and structural guidance for survival and regeneration [49]. Peripheral nervous system is capable of spontaneous regeneration due to the intrinsic regenerative capacity. However, gap injuries and chronic nerve injuries, in particular, require exogenous trophic and topographical support for effective regeneration [66,67]. Nerve tissue engineering holds promise for developing a viable new strategy subjective to the availability of effective neurotrophic cell sources [68]. MSC derived from variety of tissues have been widely known to possess multilineage capacity and multifunctional potency [28,37,51,68]. The ability of these cells to support the trophic needs of the neuronal cells for survival, development, and plasticity can be defined as “neurotrophic capacity” within the context of functional multipotency of the stem cells. ASC release a variety of neurotrophins and support neuronal survival and axonal outgrowth, suggesting their neurotrophic activity [37,68]. Furthermore, several studies reported the potential of ASC for supporting the neuronal survival and axonal regeneration by releasing a variety of growth factors, cytokines, extracellular matrix (ECM) molecules, and miRNAs [69]. Thus, the potential use of ASC for treating neurological disorders and injuries gained significant clinical interest, although the trophic capacity of ASC still remained inferior to SC [51,68].

The neurotrophic potential of ASC lies within the secretome containing a variety of biochemical and molecular factors [28,29,44,56,69]. ASC exosomes with miRNA21, miRNA222, and miRNAlet7a support neuronal survival by inhibiting apoptotic pathways. In addition, the secretome of ASC contains wide range of growth factors, i.e., nerve growth factor (NGF), glial cell-derived neurotrophic factor (GDNF), brain-derived neurotrophic factor (BDNF), neurotrophin-3 (NT3), insulin-like growth factor 1 (IGF-1), vascular endothelial growth factor (VEGF), epidermal growth factor (EGF), basic fibroblast growth factor (bFGF), transforming growth factor beta (TGF-β), and platelet-derived growth factors (PDGF) [35,38,39,69,70,71,72]. Furthermore, transplanted ASC may activate the endogenous SCs, resulting in enhanced recruitment to the injury site [31].

Within this context, we hypothesized important changes in the neurotrophic capacity of ASC in response to exogenous NTF stimulation. Therefore, the present study was designed to test the neurotrophic capacity of ASC following exogenous NTF stimulation. For this, we have primarily evaluated NTFs representing three different families, i.e., the neurotrophins (NGF, BDNF, NT3, and NT4), the GDNF family of ligands (GDNF), and the neurotrophic cytokines (CNTF), for ex vivo stimulation of ASC. The resulting condition medium (CM) as well as individual NTFs were investigated separately for their capacity to promote axonal outgrowth using DRG explants in vitro.

The cellular and molecular biology of the different species are highly conserved [73,74] and further, the structural homology of the various growth factors between human and chicken is more than 80% [75,76]. Moreover, several studies have demonstrated effective axonal outgrowth from chick DRG explants in response to human NTFs [60]. The limitation of DRG model is that it only considers sensory neurons. DRG explants possess many advantages such as easy accessibility, handling, modest costs, and three-dimensional cellular contact between neuronal and non-neuronal cells. Although the injury-induced changes are largely common among various stages of the neurons, embryonic neurons possess higher plasticity than the adult neurons [51,58,59,60,66]. However, several studies have shown that injury-induced expression of various growth factors and surface receptors that are responsive for both adult and embryonic neurons [37,77]. Thus, our in vitro model involving biological materials of different species should provide relevant information on the neurotrophic capacity of the secretome derived from ASC after differential stimuli.

To build a scientific rationale using a multimodal system, it is extremely important to consider the compatibility of the study design with the associated principles of concerned disciplines, i.e., stem cell biology, neurobiology, pharmacology, and toxicology. For this, there were set of guidelines, i.e., non-clinical safety evaluation of drug and biologic combinations published by European Medical Agency (EMA) as well as food and drug administration (FDA) [78]. In compliance with the above-indicated guidelines, our multimodal system represents a valuable in vitro model for basic and translational studies.

NGF is a well-studied neurotrophic factor due to its role in neuronal survival and axonal regeneration. In our assay, NGF clearly elicited abundant axonal outgrowth with strong branching, as reported earlier [60]. This effect is caused by NGF’s specific binding to the high-affinity TrkA receptor of sensory and sympathetic neurons [79]. GDNF, through its binding to the high-affinity GFRα complex [17], promoted relatively longer axonal projections in contrast to NGF. GDNF has been proven to exert trophic effects on injured motor and sensory axons [80]. This observation is supported by its upregulation in the ventral and dorsal root following injury [81]. The diverse functions of BDNF are mainly mediated by the TrkB receptor [45], and it was found to be especially elevated in sensory nerves after injury [82]. BDNF, interestingly, promoted axonal elongation as well as branching, as reported earlier [21,83,84,85,86]. In our assay, NT3 appeared to be effective for triggering axonal outgrowth, with significantly longer axonal projections. This observation is supported by previous reports, where its administration in vitro prevented sensory neuronal loss [23,87]. NT3 exerts its action by binding to the high-affinity receptor TrkC. CNTF and NT4 showed minimal enhancement of axonal outgrowth in terms of axonal length as well as axonal area, which is comparable to DRG explants cultured in growth medium alone. Godinho et al. evidenced low axonal density for CNTF grafts in vivo through binding to CNTFRα receptors. Downregulation of CNTF expression after nerve injury [88] may indicate its less pronounced role in regeneration. NT4, on the other hand, binds to the TrkB receptor in motoneurons [89] and specifically improves the functional reinnervation of slow motor units [90].

Interestingly, all NTF used for the ex vivo stem cell stimulation resulted in improved trophic potency of ASC for promoting axonal regeneration. In particular, NT3-stimulated ASC (CM-NT3-ASC) in comparison to all other NTF, elicited robust axonal outgrowth. When compared to NGF, it becomes evident that the increased axonal area did not mainly originate from axonal branching, since NT3-induced axonal projections are linearly aligned and well organized, but most probably from increased neuronal survival. The role of NT3 in preventing neuronal loss following injury has been well documented [23,87,91,92]. ASC have been shown to express receptors for various NTF [38,53,54,55,56]. Thus, the exogenous NTF stimuli may amplify the neurotrophic capacity of ASC through their receptor binding, leading to enhanced expression of regeneration-associated molecules, cytokines, and growth factors. Interestingly, a phosphoproteomic analysis revealed significant changes taking place in the phosphorylation of different proteins involved in growth factor signaling, cytokine release, stem cell population maintenance, and differentiation. In particular, FRMD8 and Notch2 showed significantly enhanced phosphorylation in NT3-stimulated ASC in comparison to unstimulated ASC.

FRMD8 mediates the controlled release of growth factors and cytokines from cells by regulating the active complex of ADAM17 and iRhom [93]. Lack of FRMD8 activity leads to degradation of ADAM17 and iRhom, resulting in low or no release of the growth factors [93]. Several growth factors, i.e., EGF receptor ligand amphiregulin (AREG), transforming growth factors alpha (TGFα), heparin-binding EGF, epigen, and epiregulin, are regulated by the coordinated function of these molecules [94,95]. Within this context, elevated phosphorylation of FRMD8 in the NT3-stimulated ASC gains significance and shows the possibility of the increased release of growth factors and cytokines. Therefore, a detailed analysis of the composition of the resulting secretome may provide more insights into the topic.

Notch2 plays a key role in the maintenance of neural progenitor cells (NPC) and differentiation [96]. Several studies reported that the mere activation of notch2 is sufficient for the maintenance and proliferation of the NPC [97,98]. On the other hand, a lack of Notch2 expression leads to differentiation of NPC into the neuronal phenotype [96]. These observations suggest that NT3-stimulated ASC might have attained endogenous changes toward neuronal lineage, but not complete differentiation. These findings are further strengthened by microscopic evidence showing the NT3-stimulated ASCs’ transition into S100 positive phenotype within 3 days, although these cells did not yet express other typical glial markers such as GFAP and p75. Furthermore, DRG explant treated with CM-NT3-ASC also exhibited significant changes taking place in the phosphorylation of different proteins involved in the actin and tubulin cytoskeletal pathways. In particular, EVL, SPEN, RIMS2 and MAP1A exhibited significantly enhanced phosphorylation in the DRG treated with CM-NT3-ASC compared to all other controls.

EVL regulates actin filament polymerization and controls a range of processes that are dependent on the actin cytoskeleton remodeling, e.g., axonal growth and extension [99]. An actin-filled growth cone precisely navigates axonal elongation, involving a dynamic process of actin polymerization and depolymerization by continuous sampling of guidance cues within the microenvironment [99]. Furthermore, netrin-1-induced filopodia formation was found to be EVL-dependent and directly correlated with EVL phosphorylation at a regulatory PKA site [100,101]. Thus, EVL proteins, a conserved family of actin-regulatory proteins, are essential for growth cone formation, dynamics of axonal growth, and elongation in response to differential guidance cues. On the other hand, MAP1A is a major component of the microtubule-linked fibrillar matrix of axons [102], regulating the microtubule cytoskeletal organization, which is crucial for axonal growth and activity-dependent remodeling of the axonal projections [102]. Within this context, SPEN is required for plasticity of the neurons, axonal outgrowth, and branching [103]. RIMS2 is known to positively regulate the dendrite arborization.

Taken together, these findings and observations at the phosphoproteomic level support the notion of enhanced trophic capacity (axonal growth) of ASC in response to NT3-stimulation.

To further analyze the molecular changes taking place in DRG explants treated with CM derived from NTF-stimulated ASC, a transcriptional analysis of the regeneration-associated genes (RAG) STAT-3 and GAP-43 was performed using qRT-PCR. For this, DRG explants treated with the most promising treatment condition, i.e., CM-NT3-ASC, were processed. The resulting data revealed a significant upregulation of RAGs. GAP-43 is upregulated in the perikaryon of neuronal cells after axotomy-induced inflammatory environment, calcium influx, and retrograde signaling of neurotrophic factors [2,7,8]. GAP-43 participates in the formation of the axonal growth cone and its associated lamellipodiae and filopodiae for axon elongation and path finding [45,46]. GAP-43 is also upregulated in denervated SC, where it supports their survival and maintenance [2,104,105]. Furthermore, GAP-43 is involved in supporting the long-term survival of denervated SC, as well as in the maintenance of the phenotype of repair SC [13]. JAK/STAT3, on the other hand, belongs to the main transcriptional mediators that encode for the expression of growth-associated genes such as GAP-43 [2,13,106]. Its transcription is activated by receptor-ligand binding and has been shown to play an important role in the initiation phase of axonal outgrowth and early regeneration response [107]. Our results illustrate the correlation between activation of the STAT-3 transcriptional pathway and upregulation of the effector RAG, i.e., GAP-43 in DRG explants treated with CM-NT3-ASC. A possible explanation for the upregulation of the STAT-3 pathway in DRG explants might be the array of growth-promoting factors within the secretome of NT3-stimulated ASC.

Therapeutic targets of damaged peripheral nerve, various subsets of neurons and glial cells require molecular, trophic, and guidance support for effective regeneration. Although peripheral nervous system is capable of spontaneous regeneration due to the intrinsic regenerative capacity, long-gap and chronic nerve injuries, in particular, require exogenous bioactives support for regeneration [49]. Clinically optimal nerve regeneration can be achieved by orchestrating a permissive microenvironment at the injury site. For this, complex array of biological factors is required [48,49]. Up-to-date, autologous nerve grafting remains the gold standard treatment for nerve gap injuries, which might be attributed to the presence of Schwann cells and structural extracellular matrix proteins [5,12]. For the future, nerve conduits combined with a cell-based therapy might be the most natural and effective way to mimic the autograft’s features [108]. Neurotrophic cells provide long-term availability of NTF in a physiological context, which includes adequate release kinetics and quantities, and feedback mechanisms regulating NTF release [109]. Moreover, transplanted stem cells create an interactive regenerative niche, not only by secreting factors but also by responding to endogenous signals. Although the results are promising, existing strategies for improving the trophic capacity of ASC, i.e., biochemical induction [39,110] or genetic induction [23,111,112], are associated with several drawbacks such as lengthy processes (more than three weeks), safety and regulatory hurdles. Moreover, several studies reported the deleterious effects of using large number of biological factors for axonal outgrowth in vivo [50]. Thus, there is need for simple yet effective strategies such as cell with improved trophic capacity that is similar to SC.

We, therefore, propose in the present study a simple and effective strategy involving ex vivo stimulation of ASC using exogenous NTF. Notably, our approach may reduce or avoid the complexity of administering additional exogenous growth factors in vivo, while maximizing the regenerative support for severed neuro-glial55 cells. Among all the NTF, NT3 exerted a remarkable stimulus to enhance the neurotrophic capacity of ASC, as evidenced by the phenotypic transition, axonal outgrowth, transcriptional, and phosphoproteomic data. NT3 quantitative measurements resulting from CM of different experiments together with the axonal outgrowth outcome indicate that ASC’s enhanced neurotrophic capacity for triggering axonal growth in vitro is NT3 dependent. Although the underlying mechanism remains to be elusive, several lines of evidence at cellular and molecular level support the endogenous changes that are linked with neuro-glial lineage of ASC after NT3-stimulation. Within this context, detailed account of composition of the secretome derived from the NT3-stimulated ASC would hold a key for further insights.

Several studies indicate that SC are the source of the NT3 in the damaged peripheral nerves, but not the axons, as evidenced by the NT3 heterozygous knockout mice displaying the decreased SCs, myelin retardation and high neuro-filament packing density of axons [52,113,114,115]. Further, NT3 is a potent factor for sensory neuronal survival and axonal regeneration [103]. Moreover, NT3 is crucial for survival of SCs following injury and mature SCs in adult nerves are able to survive in the absence of axons by establishing an autocrine survival loop [52]. Thus, the NT3 appears to be one of the key factors of the autocrine loop that supports long-term SC survival [52,115], nerve regeneration, and remyelination after peripheral nerve injury [115]. However, chronic nerve injuries result in SC atrophy and reduced expression of receptors and growth factors by SC [113,114]. Given these facts, NT3 cross-talk with DRG neurons appears to be one-way.

Thus, the data and knowledge obtained in the present study forms strong basis for the further refinement of the therapeutic strategy and evaluation in animals (immune-deficient nude rats), i.e., tracking/analysis of transplanted cells, anatomical, behavioral, electrophysiological recovery, and re-innervation.

## 5. Conclusions

In the present study, we demonstrated the feasibility of modulating ASC’s neurotrophic potential in vitro as evidenced by axonal outgrowth, transcriptional, and phosphoproteomic data. In particular, the conditioned medium resulting from NT3-stimulated ASC triggered robust axonal outgrowth with significantly longer projections. Together, these findings and observations provide new knowledge that should be relevant to ASC biology, basic neurobiology, and nerve repair. Further, these findings need to be validated using adult neurons and translated into a rat model of nerve-gap injury for evaluating the biology of the NT3-ASC and spatiotemporal function.

## Figures and Tables

**Table 1:** Quantitative measurements of the axonal outgrowth resulting from DRG explants treated with differential stimuli. Data expressed in mean ± SD of *n* = 12.

Group	Axonal Length (μm)	Axonal Area (mm^2^)
NGF	413 ± 182	1.903 ± 1.239
GDNF	405 ± 116	1.252 ± 0.486
BDNF	419 ± 73	1.566 ± 0.479
CNTF	352 ± 74	0.766 ± 0.286
NT3	463 ± 121	1.819 ± 0.700
NT4	291 ± 51	0.674 ± 0.123
GM	282 ± 41	0.616 ± 0.125
CM-ASC	354 ± 31	0.999 ± 0.296
CM-NGF-ASC	526 ± 87	2.460 ± 0.586
CM-GDNF-ASC	505 ± 75	1.512 ± 0.334
CM-BDNF-ASC	598 ± 118	1.918 ± 0.547
CM-CNTF-ASC	599 ± 58	2.032 ± 0.643
CM-NT3-ASC	765 ± 134	3.423 ± 0.798
CM-NT4-ASC	522 ± 80	1.617 ± 0.427

## Supplementary Materials

The following are available online at https://www.mdpi.com/2073-4409/9/9/1939/s1, Figure S1: Characterization of human adipose-derived stem cells by immunocytochemistry, Figure S2: Characterization of NT3-stimulated human adipose-derived stem cells by immunocytochemistry, Figure S3: Frequency plots of *p*-values obtained for DRG explants with different treatment conditions compared to control GM, Figure S4: Quantitative phosphoproteomics analysis, Table S1: Phosphoproteomic data obtained from stimulated-ASC and unstimulated-ASC, and Table S2: Phosphoproteomic data obtained from DRG explants treated with differential stimuli.
