# Supplementary figures and images for: Modulation of Human Adipose Stem Cells’ Neurotrophic Capacity Using a Variety of Growth Factors for Neural Tissue Engineering Applications: Axonal Growth, Transcriptional, and Phosphoproteomic Analyses In Vitro
Source: Cells. 2020 Aug 21;9(9):1939. doi: 10.3390/cells9091939 (PMC7565501; doi:10.3390/cells9091939)

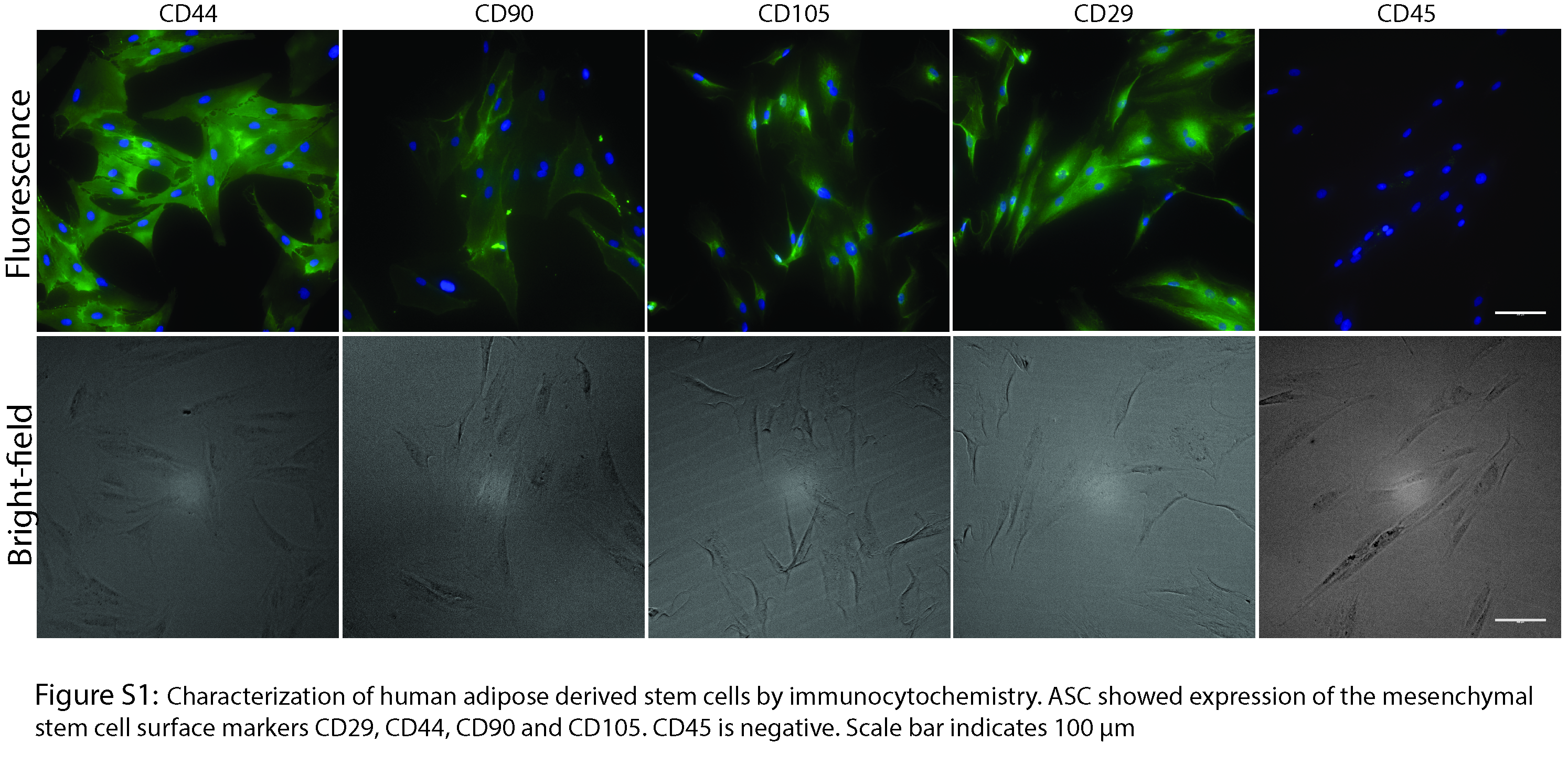

Supplement: Supplementary file 1 [file cells-09-01939-s001.zip › Figure S1.tif]

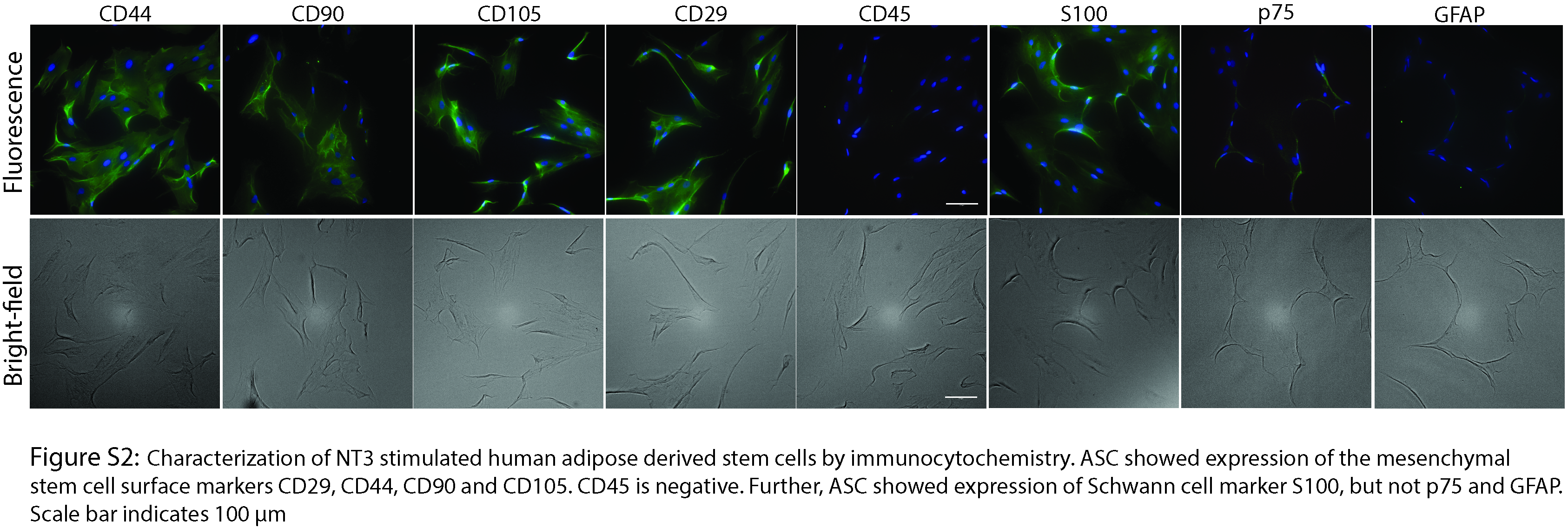

Supplement: Supplementary file 1 [file cells-09-01939-s001.zip › Figure S2.tif]

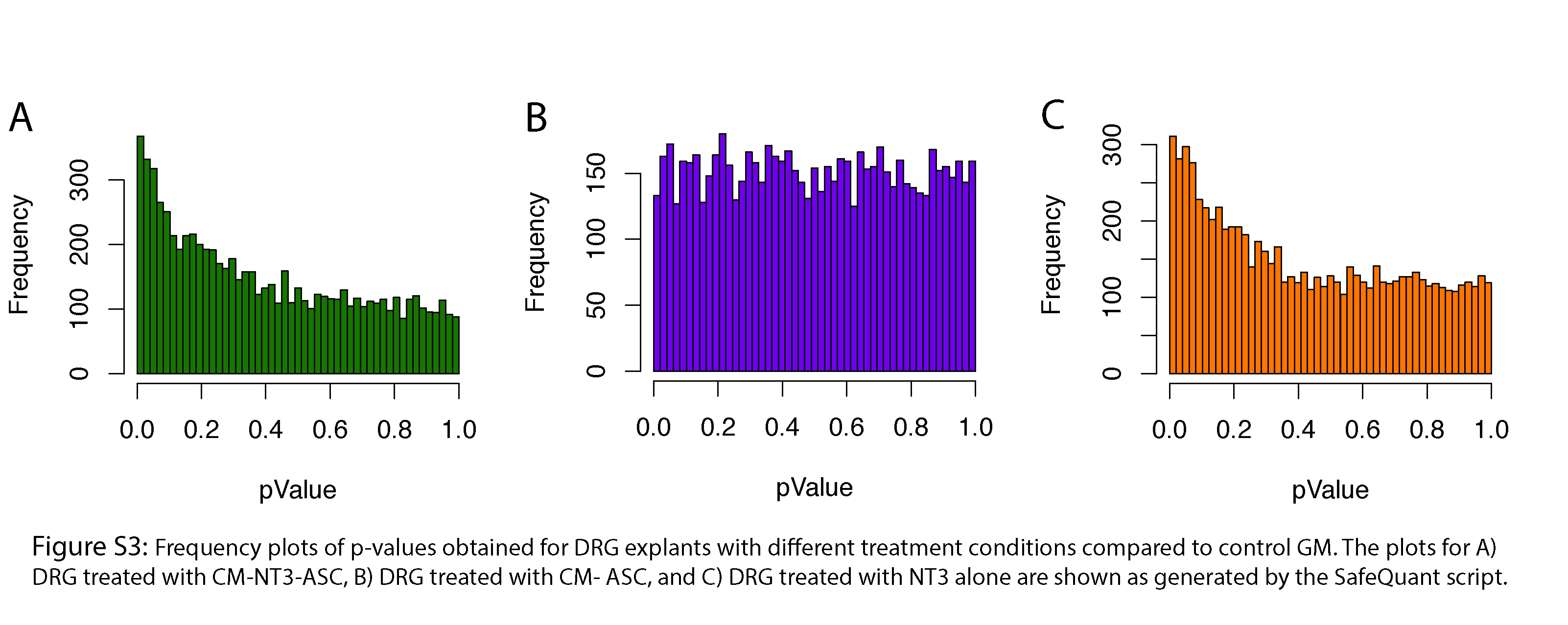

Supplement: Supplementary file 1 [file cells-09-01939-s001.zip › Figure S3.tif]

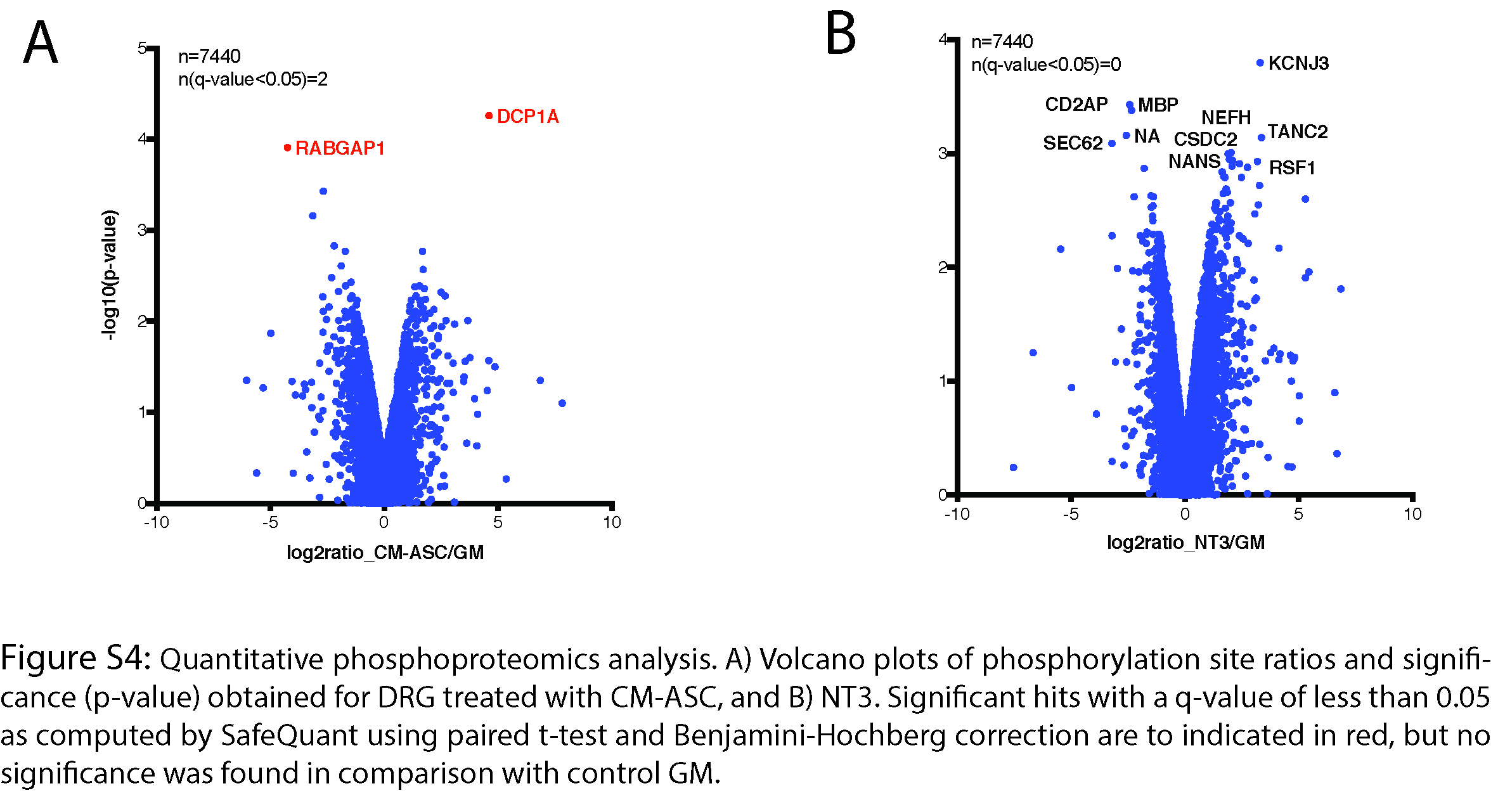

Supplement: Supplementary file 1 [file cells-09-01939-s001.zip › Figure S4.tif]
